# Changes in the Composition, Antioxidant Activity, and Sensory Attributes of Olive Oil Used as a Storage Medium for Dried Tomato Preservation

**DOI:** 10.3390/molecules29235497

**Published:** 2024-11-21

**Authors:** Dora Klisović, Anja Novoselić, Karolina Brkić Bubola

**Affiliations:** Institute of Agriculture and Tourism, Karla Huguesa 8, 52440 Poreč, Croatia; klisovic.d@gmail.com (D.K.); novoselic.anja@gmail.com (A.N.)

**Keywords:** olive oil, phenolic compounds, volatile compounds, antioxidant activity, storage, sensory attributes, shelf-life

## Abstract

The presence of food in extra virgin olive oil (EVOO) during simultaneous storage might bring additional changes to the oil’s composition and quality. To investigate this matter, the influence of dried tomatoes on the oxidative and hydrolytic parameters, fatty acids, phenolic and volatile composition, antioxidant activity, pigments, and sensory attributes of EVOO during six months of simultaneous storage at room temperature (RT; 22 ± 2 °C) and +4 °C, was studied. Lower storage temperature reduced the hydrolytic and oxidative degradation of oils when dried tomatoes were immersed. The dried tomatoes addition did not affect the fatty acids composition of EVOO. The accelerated degradation kinetics of individual phenolic compounds and antioxidant activity of oils were influenced by the presence of dried tomatoes, being more pronounced at RT. C6 and C5 volatiles responsible for the green odour of EVOO decreased, while tomato-derived volatiles (3-methylbutanal and acetic acid) increased during simultaneous storage with dried tomatoes, especially at RT. The addition of dried tomatoes diminished the intensities of EVOO’s positive attributes (fruitiness, bitterness, and pungency) while enhancing the tomato odour attribute. This study demonstrates that the introduction of dried tomatoes significantly alters the composition and quality of EVOO when used as a storage medium.

## 1. Introduction

Extra virgin olive oil (EVOO) is appreciated for its pleasant flavour and for its healthy and nutritional properties, mainly associated with its high monounsaturated oleic acid content. In addition to health benefits, the fatty acid composition is also responsible for EVOO’s high quality and oxidative stability, mostly related to alteration due to susceptibility to oxidative degradation [1,2]. It provides good resistance and extends the quality of EVOO during prolonged storage conditions [3,4] and gives it strong resistance to high temperatures [5,6]. The single double bond present in the monounsaturated fatty acid (MUFA) structure provides resistance to oxidation when compared to polyunsaturated fatty acids (PUFAs), which are prone to oxidation [2,7].

Although present in small amounts, the minor fraction of EVOO plays an important role in virgin olive oil’s quality and oxidative stability, along with the fatty acid composition abundant in monounsaturated oleic acid [2,8]. The minor, unsaponifiable fraction of EVOO consists of numerous compounds that include tocopherols, pigments (chlorophylls and carotenoids), hydrocarbons (squalene), sterols, terpene alcohols, and phospholipids [9]. Biologically active compounds, such as phenolic compounds, and a plethora of volatile compounds are also present and contribute to its high nutritional value and pleasant sensory characteristics [10,11]. These components are related to EVOO’s quality in terms of health properties, oxidative stability, and sensory properties [12]. They are also responsible for EVOO’s quality in terms of shelf life, mainly related to alteration due to susceptibility to oxidative degeneration [1]. Secoiridoid derivatives, as the most abundant phenolic compound found in EVOO, are directly responsible for the bitterness and pungency of EVOO [1,12]. EVOO is susceptible to different alterations during its storage. Hydrolytic and oxidative degradation are the main causes of the formation of undesirable products that directly influence the oil’s quality and composition [1,13], and the rate of degradation depends mainly on the composition of the oil as well as on the storage time and conditions [2,4].

The addition of food to the EVOO during storage might bring additional changes to the oil’s composition and quality. In the Mediterranean diet, EVOO is most commonly used as a dressing or as a medium to preserve different kinds of food, such as vegetables, fish, or dairy products, in order to reduce food’s exposure to oxygen. Despite the various applications on different food products and the probability of the transfer and interaction of ingredients, there is only a small amount of research on this issue [14,15,16,17]. This issue was investigated in different foods, for example, vegetables [18,19], strained yogurt [15,17], and cheese [14], under real storage conditions in only a few studies. Sicari et al. [19] evaluated the shelf-life of “Sun Marzano” dried tomato slices preserved in EVOO and reported enrichment of the EVOO due to the phytochemicals contained in dried tomatoes. However, this study did not consider the individual phenolic and volatile content of the oil used, which are responsible for its odour, taste, and nutritive value, or the influence of dried tomatoes on the sensory characteristics of oil used as a storage medium.

Therefore, in the present study, the influence of dried tomatoes on the oxidative and hydrolytic parameters, fatty acids, phenolic and volatile composition, antioxidant activity, pigments, and sensory attributes of monovarietal Istarska bjelica (IB) cultivar EVOO during six months of simultaneous storage at two different storage temperature was studied. This study is the first to examine how dried tomatoes affect the stability of biologically active phenolic compounds as well as the volatile compounds and sensory characteristics of EVOO used as a preservation medium.

## 2. Results and Discussion

### 2.1. Quality Parameters

Basic quality parameters (FFA—free fatty acids; PV—peroxide value; K_232_, K_268_, and ΔK—spectrophotometric indices) were analyzed in all the oil samples to determine the influence of dried tomatoes on EVOO’s hydrolytic and oxidative status during storage (Table 1). Quality parameters, PV, and spectrophotometric indices provide information regarding the oxidation status of the oil as standard indicators of quality regulated by the European Commission [20]. Two of the factors that influences the acceleration of EVOO’s oxidative degradation during storage are high temperatures and light [2,21]. The storage proceeded in darkness at two temperatures: RT and the temperature of refrigeration of 4 °C to study the influence of the storage temperature and the presence of dried tomatoes.

The FFA content indicates the hydrolytic degradation of lipids during storage. Considering the storage of EVOO with dried tomatoes, slight changes in the FFA values during the storage period indicated that there was no extensive hydrolytic degradation in EVOO during six months of storage under the investigated conditions (Table 1). According to the FFA value, all investigated olive oils, with or without dry tomato addition, remained within the limits prescribed for the EVOO category [20]. However, there was a statistically significant but minor enhancement of FFA formation in the EVOO + T (RT) samples after three months of storage, which was more pronounced after six months (Table 1). This indicates that the addition of dried tomatoes influenced a more rapid generation of FFA at RT compared to the control (EVOO) and EVOO + T stored at 4 °C. In a study by Sicari et al. [19], it was observed that after 12 months of storage at RT, the dried tomatoes resulted in a slight inhibition of the hydrolytic degradation in EVOO, which is not in concordance with the results of this study. The reason for the observed difference could lay in the higher initial levels of FFA in the EVOO used in the study by Sicari et al. [19] (0.68%) compared to this study (0.13%, Table 1). Such high levels of FFA could have caused a higher degradation in the control EVOO stored without the addition of dried tomatoes, and therefore, the differences in the conclusions. In our previous study [14], it was noted that a higher amount of moisture in cheese correlated with the accelerated formation of FFA during two months of storage in EVOO. The presence of water introduced by the cheese facilitated moisture exchange between the two food matrices [16], which led to a more rapid generation of FFA. Such notable changes were not observed in EVOO when the dried tomatoes were immersed (Table 1), probably due to a small amount of moisture that dried tomatoes contain (≤20%). This can be attributed to the major role of the moisture content of food in accelerating FFA formation in EVOO during simultaneous storage. The stimulation of hydrolytic degradation by the water content has already been observed under cooking conditions [22]. Considering the oxidative indicators, after one month of storage, it was noted that the values of PV and K_232_, as indicators of primary oxidation, were the highest in the control EVOO stored at RT, indicating the start of primary oxidative degradation, whereas other samples showed similar levels remaining mostly unchanged (Table 1).

A similar trend was noted during the entire period of storage which indicated that the addition of dried tomatoes resulted in slower oxidative degradation of the oil. After six months of storage, the values of K_232_ and K_268_ emerged beyond the level for the EVOO category [20] in the control EVOO stored at RT, indicating enhanced oxidation and formation of both primary and secondary oxidation products, indicated by K_232_ and K_268_, respectively, which was not the case in oils stored with tomatoes and the ones stored at the lower temperature (Table 1). This was expected considering that linear increase in quality indices (PV, K_232_, and K_268_) during 21 months of storage in darkness at RT was previously reported [4]. K_232_ was also underlined as the most reliable indicator of olive oil quality considering that it has been noted as the first one to exceed the legal category limits of virgin olive oils stored in darkness at RT [4,23]. Furthermore, the addition of dried tomatoes in the EVOO stored at 4 °C resulted in a lower PV after three and six months of storage compared to the control EVOO (Table 1). In general, at both storage temperatures, the oxidative degradation was slower in EVOO samples stored with the presence of dried tomatoes compared to the control (Table 1), which could be prescribed to the high content of bioactive compounds present in tomatoes (e.g., carotenoids, phytosterols) with strong antioxidant activities [24] that could have slowed the oxidative degradation in the corresponding oils. Therefore, it can be stated that the oxidative indicators, PV, and spectrophotometric indices were significantly influenced by the presence of dried tomatoes (Table 1). The observed corresponds with the results obtained by Sicari et al. [19], where the presence of dried tomatoes was found to reduce the degradation of EVOO during storage in the dark as indicated by the PV. The ΔK remained unchanged during the entire storage period, regardless of the addition of tomatoes (Table 1), which is in concordance with Sicari et al. [19], where the first signs of alteration in ΔK were observed after eight months. This outcome differs from the one obtained when cheese was immersed in EVOO [14], which could be attributed to the higher content of bioactive compounds present in dried tomatoes compared to cheese [24], which reduced the oxidative degradation of the oil, indicating the importance of the chemical composition of the stored food.

Generally, lower storage temperature reduced both hydrolytic and oxidative degradation in oils observed when dried tomatoes were immersed (Table 1). The storage at lower temperatures could prolong the shelf life by inhibiting both hydrolytic and oxidative degradation in EVOO used as a storage medium for food preservation. The observed is in accordance with previous studies where temperatures lower than RT have been recommended for EVOO to maintain a high oil quality and prolonged shelf life [21,25].

### 2.2. Fatty Acid Composition

The fatty acid composition was monitored during the storage of EVOO with immersed dried tomatoes to evaluate the effect of the food addition on the EVOO’s fatty acid composition. After storage with dried tomatoes, minimal changes in the fatty acid composition noted in EVOO were predominately under the influence of the storage temperature, with no direct influence of the dried tomatoes’ addition (Table 2). The slight changes caused by the storage temperature are not in agreement with previous studies, where no changes were observed even after 36 [26] and after 28 [27] months of storage at room temperature in darkness. Considering the slight changes in fatty acid saturation, it can be concluded that measuring the absorbance at 232 nm is a more reliable tool to measure the oxidation in oils stored in darkness, which has been previously underlined [4,23].

Despite the fatty acid content of the tomato pomace (skins and seeds of the fruit), containing 50% linoleic acid, 20% oleic acid, and 15% palmitic acid [28], and of fresh tomatoes, containing 45% linoleic acid, 25% oleic acid, and 18% palmitic acid [29], significant increases in the amount of these specific fatty acids due to the presence of dried tomatoes were not detected (Table 2). It might be assumed that the 0.9% of total fat present in dried tomatoes was not sufficient to influence an observable migration during a storage period of six months. On the contrary, the migration of fatty acids between EVOO and dry tomatoes during simultaneous storage was reported by Sicari et al. [19]. It could be that the reported changes in the fatty acid composition might be related to the degradation and changes in unsaturation in the oil, without being solely related to the fatty acids’ migration. Therefore, the noted divergence could lay in the initial composition of the EVOO used in the study by Sicari et al. [19], which was of lower initial quality compared to the EVOO used for the storage of dried tomatoes in this study.

### 2.3. Phenolic Compounds

**Figure 1:**
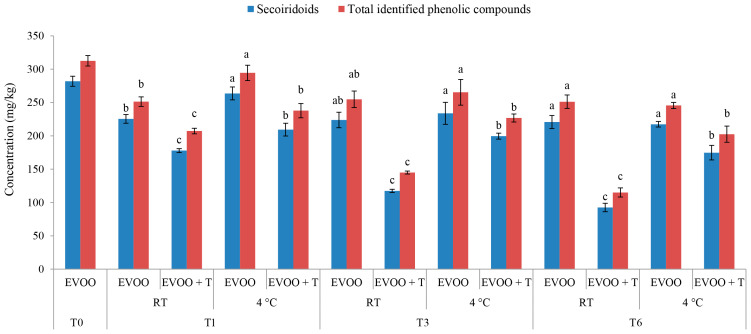
Concentration of secoiridoids and total identified phenolic compounds in fresh (T0) extra virgin olive oil (EVOO) and after one (T1), three (T3), and six (T6) months of storage with and without the addition of dry tomatoes (T) stored at room temperature (RT) and 4 °C. Results are presented as mean values ± standard deviations from three independent replicates, calculated as a sum of individual phenolic compounds of secoiridoids (3,4-DHPEA-EDA, oleuropein aglycones, ligstroside aglycones, p-HPEA-EDA). Columns labelled with a different letter within the single storage time (0, 1, 3 or 6) are statistically significantly different (Tukey’s test, *p* < 0.05).

The phenolic compounds were monitored during the storage of EVOO to investigate the influence of dried tomatoes. The total identified phenolic compounds (TIPCs) of 313 ± 8 mg/kg detected in IB EVOO used as a storage medium for dried tomatoes decreased by 63% and 35% after six months of storage at RT and 4 °C, respectively (Figure 1). The accelerated degradation kinetics of phenolic compounds in oils was influenced by the presence of dried tomatoes. This is not in concordance with the results of Sicari et al. [19], who reported significant gradual increases in the total phenolic content measured spectrophotometrically using the Folin–Ciocalteu reagent in EVOO stored with the addition of dried tomatoes in darkness. The dried tomatoes were identified as the source of the detected phenolic compounds in the analyzed EVOO, indicating the migration of tomato phenols into the oil phase [19]. It might be that the phenolic compounds from the tomatoes were extracted in the EVOO and detected spectrophotometrically, which was not possible with the high-performance liquid chromatography with diode-array detection (HPLC-DAD) method used in this study. The differences in the results from the two studies could also be justified by the general recognition of the Folin–Ciocalteu reagent’s low specificity, which could react with other compounds besides phenols [30,31]. Also, the HPLC-DAD used ensures higher sensitivity and accurate quantification [32]. Further, the results of Klisović et al. [14] indicated the significant role of the food’s initial composition. Considering that the dried tomatoes used in this study consisted of a considerable amount of proteins (8 g per 100 g of dried tomatoes), and knowing that phenolic compounds form complexes with other food components, such as proteins [33], these interactions might have led to apparent losses in the TIPC due to the inability of the HPLC-DAD method to determine particular phenolic compounds in such complexes.

After the first notable decrease in TIPC in the control EVOO stored at RT, its content over the next five months remained mostly preserved. This trend can be observed as the lack of differences in the control EVOO stored at both temperatures after three and six months (Figure 1), which indicated that the storage temperature had no significant effect on the TIPC of the control oil. Similar results were obtained in a previous study where after 12 months of storage, there were no significant differences in the total phenolic content of monovarietal Buža EVOO stored at RT and 4 °C [25]. Regarding the TIPC concentrations in EVOO + T samples at the two storage temperatures, there was a significant difference, being even more pronounced after storage at RT (Figure 1). The same trend was noted after three and six months of storage. This indicates that a higher storage temperature, despite having no influence on the control EVOO, accelerated the degradation kinetics in oils stored with the addition of dried tomatoes. Therefore, it could be stated that the addition of dried tomatoes significantly decreases the TIPC, and that at a higher storage temperature (RT), the degradation of phenolic compounds is even greater.

The composition of secoiridoids, the most abundant group of phenolic compounds, has been directly correlated with EVOO’s shelf life during storage [23]. The secoiridoids suffered significant changes in EVOO stored with immersed dried tomatoes (Figure 1). These changes were similar to the ones described for TIPC, considering that secoiridoids represented 91.1% of the initial TIPC (Table 3). After just the first month, the concentration of the two most abundant secoiridoids, oleacein (3,4-DHPEA-EDA) and oleocanthal (p-HPEA-EDA), were significantly lower in EVOO + T samples stored at RT compared to the control oil and EVOO + T stored at 4 °C (Table 3). Among other secoiridoids, the level of oleuropein, oleuropein aglycones, and ligstroside aglycones was also affected by the addition of dried tomatoes, especially at RT (Table 3). The results of the FFA indicated the highest rate of hydrolysis in EVOO + T samples stored at RT (Table 1), which correlated with the present results for phenolic compounds, indicating the highest level of hydrolytic degradation of secoiridoids in the EVOO + T (RT) samples (Table 3). After one month of storage, oleacein suffered the most notable decrease of 69.8% in EVOO + T (RT) compared to its initial concentration, and at the end of six months of storage, almost a complete reduction of 90.6% was noted (Table 3). Oleacein is most likely the secoiridoid most prone to oxidative degradation with high reactivity, mostly due to the two hydroxyl groups in its phenolic ring and two aldehyde groups in its structure. Consequently, oleacein could have reacted with other biological molecules and phenolic compounds derived from dried tomatoes, forming complex structures that interfered with the HPLC detection, which could have led to appetent reductions in its concentration. De Toffoli et al. [34] studied oleacein extracted from functionalized foods enriched with phenolic extracts and found its strong binding affinity to proteins, which could be one of the reasons for some of the observed losses.

**Figure 2:**
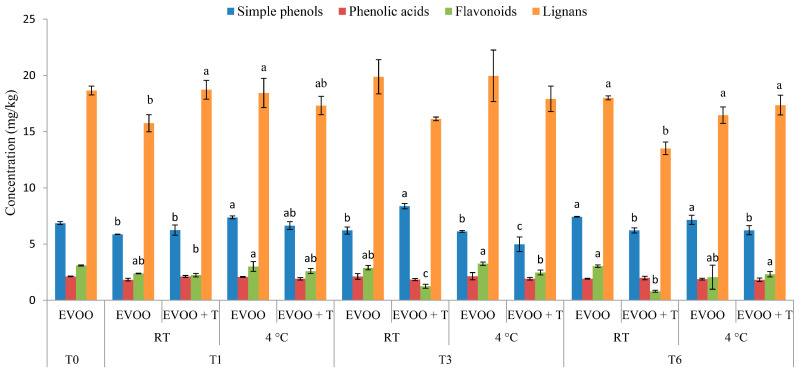
Concentration of simple phenols, phenolic acids, flavonoids, and lignans in fresh (T0) extra virgin olive oil (EVOO) and after one (T1), three (T3), and six (T6) months of storage with and without the addition of dry tomatoes (T) stored at room temperature (RT) and 4 °C. Results are presented as mean values ± standard deviations from three independent replicates, calculated as a sum of individual phenolic compounds as stated: simple phenols (hydroxytyrosol, tyrosol, vanillin, hydroxytyrosol acetate); phenolic acids (vanillic acid, p-coumaric acid); flavonoids (luteolin, apigenin); and lignans (pinoresinol, acetoxypinoresinol). Columns labelled with a different letter within the single storage time (0, 1, 3 or 6) are statistically different (Tukey’s test, *p* < 0.05).

The concentration of simple phenols, tyrosol, and hydroxytyrosol in control EVOO was found to be unchanged or even slightly increased (Table 3). Both tyrosol and hydroxytyrosol are known to increase in concentration during storage in darkness due to the hydrolysis of secoiridoid aglycones [4,35]. After six months of storage, the decrease in tyrosol concentration in EVOO + T stored at RT and 4 °C was not significantly different, which indicates that the storage temperature did not influence the tyrosol concentration and that the decrease can be attributed solely to the dried tomatoes’ addition. Additionally, the concentration of hydroxytyrosol was significantly lower in EVOO + T stored at RT compared to the one stored at 4 °C, which indicates that the higher storage temperature decreased the hydroxytyrosol concentration at a higher rate. This was not unexpected considering that hydroxytyrosol is more susceptible to oxidation compared to tyrosol due to the additional hydroxyl group present in its structure. Among the phenolic acids, p-coumaric acid increased after six months only in EVOO + T stored at RT, whereas the vanillic acid concentration decreased in the same sample (Table 3). Based on this observation, there were no differences in the total phenolic acid content between samples during storage (Figure 2). Flavonoids and lignans were also affected by the addition of dried tomatoes (Figure 2). However, slight but notable decreases were detected in EVOO + T stored at RT (Table 3).

The level of hydroxytyrosol acetate increased gradually in EVOO + T samples stored at both temperatures during the storage period while remaining unchanged in the control EVOO. The increase was even more notable in the control oil stored at RT (Table 3). Interestingly, among the phenolic compound determined, only the concentration of hydroxytyrosol acetate increased in EVOO stored with different types of cheese type [14], and such increases were correlated with the formation of acetate derived from the fermentative pathway of whey cheese [36]. However, hydroxytyrosol acetate might be useful as a tool for monitoring oxidative degradation during storage.

### 2.4. Radical-Scavenging Activity

**Figure 3:**
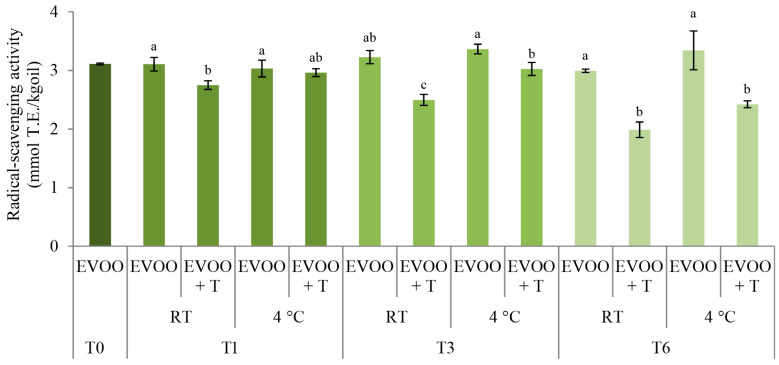
Radical-scavenging activity (mmol T.E./kgoil) in fresh (T0) extra virgin olive oil (EVOO) and after one (T1), three (T3), and six (T6) months of storage with and without the addition of dry tomatoes (T) stored at room temperature (RT) and 4 °C. Results are presented as mean values ± standard deviations from three independent replicates. Columns labelled with a different letter within the single storage time (0, 1, 3, or 6) are statistically significantly different (Tukey’s test, *p* < 0.05).

The radical-scavenging activity (RSA) potential of samples stored with the addition of dried tomatoes was investigated, and the results are summed up in Figure 3.

Decreases in the ability of the oil to inhibit the 2,2-diphenyl-1-picrylhydrazyl (DPPH) radical were noted already after one month of storage in EVOO + T (RT) samples (Figure 3). During storage, the RSA in EVOO + T (RT) gradually decreased with the storage time. After three months of storage, decreases in RSA were observed in EVOO + T stored at 4 °C compared to the control oil stored in the same conditions (Figure 3). The RSA in the control oils was mostly preserved during the entire storage period, regardless of the storage temperature, and it was higher in the oil stored with the dried tomatoes under the same storage conditions (Figure 3). This indicates that the results of the radical-scavenging activity of EVOO imply significant decreases in the antioxidant activity of EVOO stored with dried tomatoes. Correspondingly, Sicari et al. [19] reported a decrease in EVOO RSA during storage with dried tomatoes. However, calculating the antioxidant capacity as IC_50_, the data implied a preserved and increased antioxidant activity due to the oil’s enrichment with bioactive compounds deriving from the tomatoes [19].

The lowest RSA was noted in EVOO stored with tomatoes at RT (Figure 3), for which the highest decrease in the TIPC content was detected (Figure 1). This observed trend in RSA is highly related to the development of the TIPC in the corresponding oil. This can be attributed to the significant correlation between phenolic compounds and the antioxidant activity of the oils. Additionally, the reductions in antioxidant attributes can be related to the protein content of tomatoes, already underlined as one of the factors reducing the phenolic content, which leads to the reduction in the antioxidant activity of phenolic compounds [37]. However, the exact mechanisms underlying this suppression, attributed to non-covalent binding, remain poorly understood [38].

Additionally, the observed correlates with the findings of Sicari et al. [19], where a decrease in EVOO RSA during storage with dried tomatoes was reported. Still, it must be underlined that a significant level of RSA is still preserved in each of the samples despite the addition of tomatoes.

### 2.5. Pigments

**Figure 4:**
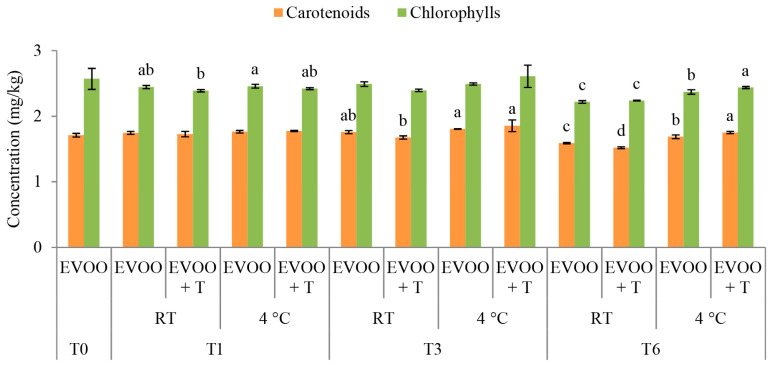
Concentration (mg/kg) of pigments in fresh (T0) extra virgin olive oil (EVOO) and after one (T1), three (T3), and six (T6) months of storage with and without the addition of dry tomatoes (T) stored at room temperature (RT) and 4 °C. Results are presented as mean values ± standard deviations from three independent replicates. Columns labelled with a different letter within the single storage time (0, 1, 3 or 6) are statistically different (Tukey’s test, *p* < 0.05).

The concentration of carotenoids during the first month of storage remained preserved with no influence related to the dried tomatoes addition (Figure 4). The chlorophylls were mostly preserved during the first three months of storage, with slight changes noted in the first month (Figure 4). After six months of storage, the concentration of chlorophylls was higher at the lower storage temperature of 4 °C. The same trend was observed for the development of the carotenoid concentration (Figure 4). Such trends are in concordance with previous research, where minimal decreases in both chlorophyll and carotenoid concentrations were detected during storage in darkness [4,26]. Similarly, the dried tomatoes resulted in a higher level of chlorophyll at the lower storage temperature, while at RT, there were no changes related to the addition of dried tomatoes (Figure 4). It must be emphasized that both carotenoid and chlorophyll concentrations remained mostly preserved during the entire storage period (Figure 4).

In a study by Sicari et al. [19], significant decreases, almost by half of the initial content of pigments, were observed after six months of storage with the addition of dried tomatoes, which differs from the results reported in this study (Figure 4). This divergence might be attributed to the quality of EVOO used in each study.

### 2.6. Volatile Compounds

The concentration of volatile compounds was determined after one and six months of storage to investigate the influence of the dried tomatoes during simultaneous storage on the compounds that are responsible for EVOO’s sensory attributes. The initial composition of volatile compounds was characterized by the predominant content of (*E*)-2-hexenal, representing 96.5% of the total volatiles (Table 4). 1-Hexanol, (*Z*)-3-hexen-1-ol, and (*E*)-2-hexen-1-ol also characterized the headspace of the analyzed IB EVOO as its main compounds, although they were present in much lower amounts. The described initial composition is characteristic of fresh and high quality EVOO [11,39].

After one month of storage, the EVOO with immersed dried tomatoes showed no changes in the total volatiles’ concentration related to the addition of dried tomatoes (Table 4). The observation indicated that after such a short storage period, total volatiles remained mostly preserved in their total concentration. Nevertheless, after six months, a significant decrease was noted in the EVOO + T samples stored at RT, whereas after storage at the lower temperature of 4 °C, there were no significant changes observed (Table 4). The results obtained for the total volatiles correlated with the results of TIPC, indicating the most notable decrease in the oils stored at RT (Figure 1). Storage at room temperature has been previously reported to promote the decomposition of specific volatile compounds during storage at RT [40]. Otherwise, high temperatures accelerate the decomposition of hydroxyperoxides formed during autoxidation, resulting in increases in certain volatiles [1,23]. However, in this study, the effects of the temperature on the two storage conditions were not noted (Table 4). The cause of the decrease in volatiles might not be completely related to oxidative degradation but to the formation of volatile compounds deriving from tomatoes and interacting with EVOO’s volatiles, which might have led to the formation of new volatile compounds in the EVOO headspace after the simultaneous storage. These volatile compounds might be undetectable with the method used, thus explaining one of the reasons for such a decreases.

The C5 volatiles, with a predominant content of 1-penten-3-one, were influenced by the addition of dried tomatoes at both storage temperatures.

However, a more pronounced degradation was observed in samples stored at RT (Table 4). This indicated that both the dried tomatoes’ presence and storage temperature had a significant influence on the C5 volatiles during EVOO storage. C5 ketones, such as 3-pentanone, have been reported to be increased in EVOO stored for several months at RT since they result from the homolytic cleavage of hydroperoxides [41]. Such increases were not observed in this study (Table 4), which might be related to the variability in the initial volatile composition differing among monovarietal oils, as indicated by several studies [41,42].

C6 aldehydes are the most abundant volatile compounds and make a significant contribution to EVOO’s flavour [35]. The C6 aldehydes decreased in concentration due to the addition of dried tomatoes after one month of storage, primarily due to the decrease in the (*E*)-2-hexenal concentration (Table 4).

The reduction was even more notable after six months, where significant decreases of 56.3% and 32.9% in EVOO + T stored at RT and 4 °C, respectively, were observed (Table 4). Even other C6 aldehydes, including (*Z*)-2-hexenal, (*E*)-3-hexenal, and (*Z*)-3-hexenal, showed a similar trend during storage, being the most decreased after storage with the dried tomatoes at RT (Table 4). Among C6 aldehydes, hexanal concentration showed a rather different development, where after six months of storage, the highest concentration was detected in control EVOO stored at RT (Table 4). This observation indicates that the presence of dried tomatoes did not decrease the hexanal concentration, but the storage temperature increased the concentration in the control EVOO stored at RT (Table 4). Considering that hexanal has been underlined as a considerable marker for rancid sensory defects related to oil oxidation [43], it might be that enhanced oxidation occurs at higher temperatures, which is also in agreement with the results obtained using quality parameters analysis in the same oil samples (Table 1).

A very weak reduction in the concentration of C6 alcohols was noted, and it was primarily influenced by the presence of dried tomatoes (Table 4). Interestingly, there were almost no significant differences between the EVOO + T samples stored at the two storage temperatures. It might be that the increase in C6 alcohols, usually related to oil degradation during storage of EVOO at RT [41], were not noted due to the influence of the dried tomatoes in the EVOO. Rather than being markers of quality and freshness [41], the contribution of C6 alcohols to the aroma of EVOO is relatively low considering the high odour threshold values [43,44].

Among other compounds, (*Z*)-2-penten-1-ol + (*Z*)-3-hexenyl acetate significantly decreased in the EVOO + T samples stored at the lower storage temperature, which was unexpected considering that (*Z*)-2-penten-1-ol was found in the volatile profile of the tomato fruits [45]. In the case of 3-methylbutanal, it is evident that the increases were solely related to the presence of the dried tomatoes (Table 4). Correspondingly, 3-methylbutanal was also found to be a compound present in the volatile profile of tomato fruit with a positive odour unit [46]. Its concentration was significantly higher in EVOO + T stored at RT, which indicated that at higher temperatures, faster release of the dried tomato volatiles occurred. Significant increases in the concentration of isoamyl acetate, (*E*)-2-octenal, and acetic acid were observed in EVOO + T samples stored at both temperatures compared to the control oils (Table 4). The increases in (*E*)-2-octenal, an unsaturated aldehyde and one of the main contributors to the rancid sensory defect in olive oils, could be related to its formation during oxidative degradation [43]. Major increases in the acetic acid concentration could be derived from the dried tomatoes since acetic acid has been found in the volatile composition of tomato paste [47]. A notably higher rate of increase in acetic acid was noted in EVOO + T samples stored at RT, which could be attributed to the previous statement that at higher storage temperatures (e.g., RT), the release of volatiles from dried tomatoes is facilitated, and oxidative degradation occurs at a higher rate.

### 2.7. Sensory Attributes

**Figure 5:**
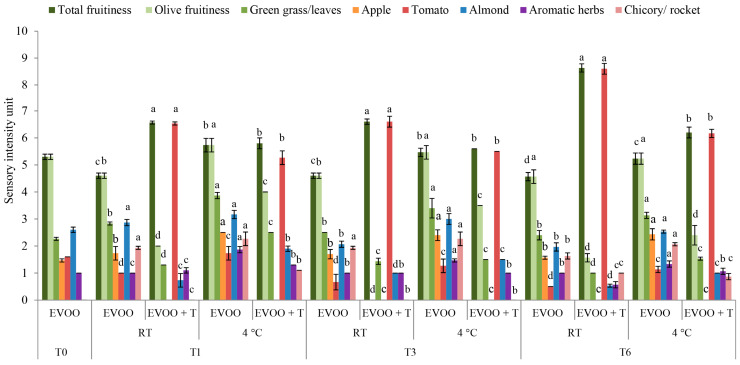
Odor attributes in fresh (T0) extra virgin olive oil (EVOO) and after one (T1), three (T3), and six (T6) months of storage with and without the addition of dry tomatoes (T) stored at room temperature (RT) and 4 °C. Results represent the mean values ± standard deviations of three repetitions. Different letters above bars within the same storage time (1, 3, or 6 months) are statistically different (Tukey’s test, *p* < 0.05) for each sensory attribute separately.

**Figure 6:**
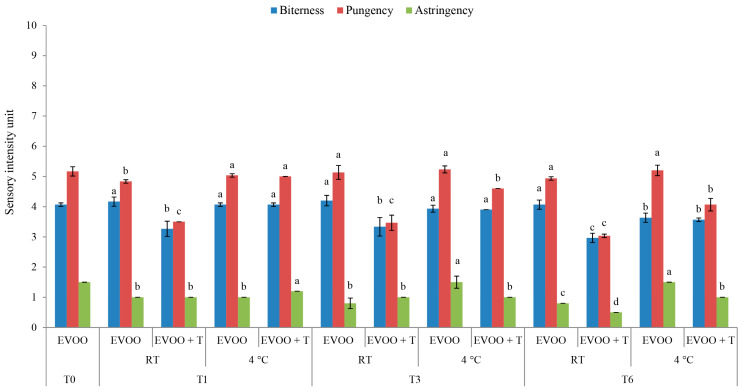
Taste attributes in fresh (T0) extra virgin olive oil (EVOO) and after one (T1), three (T3), and six (T6) months of storage with and without the addition of dry tomatoes (T) stored at room temperature (RT) and 4 °C. Results represent the mean values ± standard deviations of three repetitions. Different letters above bars within the same storage time (1, 3, or 6 months) are statistically significantly different (Tukey´s test, *p* < 0.05) for each sensory attribute separately.

The sensory attributes of oils during storage with and without the addition of dried tomatoes were determined. The results of the odour attributes are presented in Figure 5, whereas taste attributes are in Figure 6. The total fruitiness evaluated by the panellist was determined as an overall odour intensity. The fresh monovarietal IB EVOO was characterized by a medium-high total fruitiness intensity (5.3 ± 0.2; Figure 5), medium bitterness intensity (4.1 ± 0.1), and pungency (5.2 ± 0.2; Figure 6). The IB EVOO was characterized by a complex odour with characteristic green grass/leaves, apple, and almond notes. This initial sensory composition can be considered typical for IB cultivar EVOOs, whose pungency was previously characterized as strong and burning [39].

Already after the first month of storage, significant changes were noted in the EVOO’s odour attributes related to both storage conditions and the addition of dried tomatoes (Figure 5). The addition of dried tomatoes increased the total fruitiness of the EVOO + T samples stored at RT compared to control oils stored at the same temperature and oils stored at 4 °C. The same trend was observed after three months of storage. Finally, after six months, the total fruitiness intensity reached the highest level in EVOO + T samples stored at RT (8.6 ± 0.4). Slightly lower values were observed after storage at a lower temperature (6.2 ± 0.2), but they were still higher compared to both control oils (Figure 5). This indicates that the overall odour intensity was increased by the addition of the dried tomatoes during storage. However, it must be underlined that the main source of the detected odour was not related to olive fruitiness. Precisely, after the first month of storage, the odour attributes of olive fruitiness were significantly decreased in the EVOO samples stored with tomatoes (2.0 ± 0.1) compared to the control EVOOs (4.6 ± 0.3) stored at RT (Figure 5). The decrease in the olive fruitiness in the samples stored with dried tomatoes was followed by a simultaneous increase in the tomato odour attribute detected after one month of EVOO + T storage, with the highest level noted after six months of storage at RT (Figure 5). This indicates that the increases in the EVOO + T fruitiness mostly originated from the tomato attribute. The observed could be correlated with the results of the volatile compounds, which showed large increases in acetic acid concentration during the storage period already after just one month (Table 4). Acetic acid is known to contribute to tomatoes’ aroma, but it also the volatile compound that occurs as a result of fermentation, giving a stale and wine-like taste to foods [47]. Additionally, acetic acid is principally responsible for the vinegary sensory defect in virgin olive oils [43]. Even though the vinegary sensory defect was not observed by the panellists, there is a possibility that it could be after a longer storage period due to the fermentation processes [47]. It was also observed by the analysis of the volatile compounds that (*Z*)-3-hexenal and 1-penten-3-one, volatile compounds with the highest odour activity values reported for IB EVOO and responsible for the green leaf, green, and apple sensory attributes [39,44], decreased significantly in EVOO + T stored at both temperatures, with the highest decrease noted at RT (Table 4). This correlated with the results obtained by the sensory analysis, where significant decreases in olive fruitiness, green grass/leaves, and apple sensory attributes were found in samples stored with the addition of dried tomatoes, with the highest decrease rate noted at RT (Figure 5).

Taken together, all the decreases in the odour sensory attributes related to the olive fruitiness, green grass, and apple attributes were more intense in the EVOO + T stored at RT compared to the lower temperature. Correspondingly, the increase in the tomato-like attribute was higher in the EVOO + T stored at RT (Figure 5). This once again correlates with the observed dynamic reported for the volatile compounds, where compounds related to the olive fruitiness (e.g., (*Z*)-3-hexenal, and (*E*)-2-hexenal) were the most decreased at RT, and ones related to the tomato sensory attribute (e.g., acetic acid and 3-methybutanal) and known to be derived from the dried tomato volatile composition were highest at RT. Again, at RT, the release of the tomato-like sensory attributes and volatiles is accelerated compared to the lower temperature, indicating that preservation of the fruitiness related to olives is achievable at lower storage temperatures.

Considering the taste attributes, a decrease in bitterness, pungency, and astringency was noted in oils stored with the addition of dried tomatoes at RT during the storage period (Figure 6). This corresponded with the results obtained by the analysis of the phenolic compounds (Table 3), where the most notable general decrease in secoiridoids was observed in the oils stored with dried tomatoes at RT (Table 3). The observed was expected considering the strong correlation between virgin olive oil pungency and bitterness with the concentration of secoiridoids in virgin olive oils [48,49]. On the other hand, the addition of dried tomatoes during storage at the lower temperature of 4 °C had no significant influence on the EVOO bitterness (Figure 6). However, a slight decrease in pungency was noted after three and six months of storage (Figure 6). Between the EVOO + T and control oil stored at 4 °C, no significant difference in the oleocanthal concentration was observed, but there was a significant difference in the concentration of oleacein (Table 3). Since oleocanthal is considered to be the key source of virgin olive oil pungency, and oleacein of bitterness and astringency [48,49], no direct correlation could be defined between phenolic compounds and the taste attributes in this regard. However, it must be considered that the noted differences in the taste attribute, while statistically significant, can be considered minimal.

## 3. Materials and Methods

### 3.1. Materials

Monovarietal Istarska bjelica olive (*Olea europaea* L.) cultivar EVOO was used as the storage medium for dried tomatoes. The IB olive fruits were manually harvested on 10 October 2019, from an orchard located on the Istrian peninsula, Croatia (44°58′ N, 13°48′ E). The fruits were in the typical ripening stage for the IB cultivar, with a ripening index of 1.10 determined by the method of Beltrán et al. [50]. A two-phase extraction system (Model SPI 222 S, Pieralisi, Iesi, Italy) was used to extract the oil samples within 24 h of harvesting. The olives were first crushed using a knife crusher to produce an olive paste. This olive paste was then malaxed for 30 min at a controlled temperature of 25 ± 1 °C in a vertical mixer, allowing for optimal oil release. Following malaxation, the oil was extracted by centrifuging the paste in a two-phase decanter, effectively separating the oil from the solids and water. After extraction, the oil was filtered using a filter press with cellulose filter plates and stored in 1 L dark green glass bottles at 22 ± 2 °C until the experiment began.

Dried tomatoes (nutritional content: 8 g of proteins, 28 g of carbohydrates, and 0.9 g of fat per 100 g of dried tomatoes; ≤20% moisture according to the nutritive declaration of the product) were purchased from a local store in Poreč, Croatia. For the experiment, 15 ± 0.1 g of previously homogenized dried tomatoes were placed into a jar filled to the top with 150 mL of IB EVOO. To prevent light exposure, samples of EVOO with dried tomatoes (EVOO + T) in jars were subsequently wrapped with aluminium foil and stored for six months at two temperatures: room temperature (RT; 22 ± 2 °C) and a refrigerator temperature of 4 ± 1 °C. Three jars per storage temperature and for each of the three time points (after one, three, and six months of storage) were prepared. Control samples in the form of jars filled to the top with EVOO without the addition of dried tomatoes were prepared for each time point and storage temperature (three jars) and stored in the same storage conditions as the study samples for six months. All oil samples (the control as well as those stored for 1, 3, and 6 months) underwent comprehensive chemical and sensory analyses at each evaluation point. However, volatile compounds were only monitored in the control samples and those stored for 1 and 6 months.

### 3.2. Quality Parameters

The oil quality parameters, free fatty acids (FFAs) [4,51], peroxide value (PV) [52], and spectrophotometric indices (K_232_, K_268_, and ΔK) [53], were measured according to the International Olive Council (IOC) analytical methods.

### 3.3. Fatty Acids Analysis

The preparation of the fatty acid methyl esters (FAMEs) from olive oils was performed by trans-esterification with a methanolic solution of potassium hydroxide at room temperature according to the standard IOC method [54] by vigorous shaking of the oil solution in heptane (0.1 g in 2 mL) with 0.2 mL of 2 M methanolic KOH in a screwcap vial. The analysis of FAMEs was also performed according to the IOC standard method [54] using a Varian 3350 gas chromatograph (Varian Inc., Harbour City, CA, USA) equipped with a flame ionization detector (FID) and an Rtx-2.330 capillary column (60 m × 0.25 mm id × 0.10 µm film thickness) (Restek, Bellefonte, PA, USA). Identification of FAMEs was based on their retention times with respect to the standard FAME mixture (Sigma, Steinheim, Germany) and according to the IOC reference method [54], and results are expressed as proportions (%) of total fatty acids.

### 3.4. Phenolic Compounds Analysis

Phenolic compounds in oil samples were extracted and analyzed following the method of Jerman Klen et al. [32], with modifications by Lukić et al. [55], using an Agilent Infinity 1.260 HPLC system (Agilent Technologies, Santa Clara, CA, USA). The phenolic compounds were identified by comparing retention times and UV–Vis spectra with those of pure standards and previously reported data [32]. The detection was carried out at 280 nm for simple phenols, lignans, secoiridoids, and vanillic acid; at 320 nm for vanillin and p-coumaric acid; and at 365 nm for flavonoids. Quantification was performed using standard calibration curves for tyrosol, hydroxytyrosol, vanillic acid, vanillin, p-coumaric acid, luteolin, apigenin, pinoresinol, and oleuropein following the chromatograms presented in Figure S1. The concentrations of phenolic compounds were expressed as mg/kg oil. Semi-quantitative analysis was performed for hydroxytyrosol acetate, acetoxypinoresinol, and secoiridoids, where the concentrations were expressed as hydroxytyrosol, pinoresinol, and oleuropein, respectively, assuming a response factor equal to one, as in Lukić et al. [55]. The concentrations of phenolic compounds were expressed in mg/kg of oil, and TIPCs were reported as the sum of all identified phenolic compounds.

### 3.5. Radical-Scavenging Activity

The antioxidant activity of the oils was assessed by measuring the free radical-scavenging activity of the DPPH radical according to the method of Koprivnjak et al. [56]. Absorbance was recorded using a Varian Carry 50 spectrophotometer (Varian Inc., Mulgrave, Victoria, Australia). A calibration curve was created with Trolox solutions of known concentrations, and results were presented as mmol Trolox equivalent (TE) per kg oil.

### 3.6. Pigments

Chlorophyll and carotenoid contents in oil samples were determined using the method described by Minguez-Mosquera et al. [57]. A 7.5 g olive oil sample was weighed and dissolved in cyclohexane, bringing it to a final total volume of 25 mL. Concentrations were calculated from the absorption spectrum; absorption at 670 nm corresponds to the chlorophyll fraction, primarily composed of pheophytin a. The primary carotenoid pigment, lutein, was measured at an absorption of 470 nm. A UV–Vis spectrophotometer (Varian Cary 50, Varian, Harbour City, CA, USA) was used for the analysis, and the results were expressed as mg/kg of pheophytin a and lutein content, respectively.

### 3.7. Volatile Compounds Analysis

Volatile compounds in oil samples were extracted using headspace solid-phase microextraction (HS-SPME) based on the method described in Brkić Bubola et al. [58,59] with modifications according to Brkić Bubola et al. [60]. The volatile compounds analysis was carried out using a Varian 3350 gas chromatograph (Varian Inc., Harbor City, CA, USA) equipped with FID and a capillary column Rtx-WAX (Restek, Bellefonte, PA, USA). To identify volatile compounds, a Varian 3900 GC was coupled to a Varian Saturn 2100 T ion trap mass spectrometer (Varian Inc., Crawley, UK) using the same column and temperature program. Compound identification was achieved by comparing retention times and mass spectra with those of pure standards and reference data from the NIST05 library. Quantification was carried out using calibration curves of pure standards [60], and semi-quantitative analysis was conducted by expressing concentrations as equivalents of structurally similar compounds, assuming a response factor of 1. The total volatile content was calculated as the sum of all identified volatile compounds.

### 3.8. Sensory Characteristics

A quantitative descriptive analysis of virgin olive oil samples was conducted according to the standard method defined by the IOC [61]. Sensory analysis was carried out by the Panel of the Institute of Agriculture and Tourism (Poreč, Croatia), which is accredited for sensory analysis of virgin olive oil [62] and recognized in continuation by the IOC from 2014. The panel consisted of eight trained assessors (five female and three male, with an average age of 40) who were experienced in evaluating virgin olive oil following the IOC method and guide [61,63]. To maintain high accuracy in sensory analysis, panel members underwent continuous training sessions, refining their qualitative and quantitative skills in line with IOC standards. Performance monitoring was implemented through ongoing internal quality control procedures [64]. All panellists provided informed consent to participate in the study. The sensory characteristics of odour and taste were quantified on an ordinal intensity scale from 0 to 10 (where 0 represents no perception and 10 represents the highest intensity). For a more detailed profile, the evaluation sheet was expanded to include specific sensory attributes: olive fruitiness, green grass/leaves, apple, tomato, almond, aromatic herbs, chicory/rocket, and astringency. Fresh oil samples were assessed in triplicate as well as at intervals of one, three, and six months, with and without dried tomato additions (three replicates per treatment, totalling 12 samples at each storage evaluation point). Before analysis, oils stored with dried tomatoes were strained to remove solid particles. A maximum of 12 samples were evaluated per day, with replicates presented in random order.

### 3.9. Statistical Analysis

Statistical significance between treatments was assessed using one-way ANOVA followed by Tukey’s honest significant difference post-hoc test for multiple comparisons of means (*n* = 3) at a significance level of *p* < 0.05. All statistical analyses were performed using Statistica software, version 13.2 (StatSoft Inc., Tulsa, OK, USA).

## 4. Conclusions

The influence of dried tomatoes on the composition of EVOO used as a storage medium at two different storage temperatures (RT and 4 °C) was investigated. The present study represents one of the rare studies that demonstrates how introducing different food products can significantly alter the chemical and sensory profile of EVOO when used as a storage medium. It was found that lower storage temperatures used for dried tomato preservation helped to reduce hydrolytic and oxidative degradation of the EVOO, potentially extending its shelf life. The presence of dried tomatoes did not affect the fatty acid profile of the EVOO used as a storage medium. However, this research is the first to report on the influence of dried tomatoes on the stability of phenolic and volatile compounds as well as the sensory attributes of EVOO used in this manner. The presence of dried tomatoes led to a decline in both the total phenolic content and specific phenolic compounds, which contributed to a noticeable reduction in the oil’s sensory qualities, particularly its taste. The degradation of phenolic compounds was accelerated at higher storage temperatures, which also resulted in a decrease in EVOO’s radical-scavenging activity during simultaneous storage with dried tomatoes. Additionally, storing EVOO with dried tomatoes altered its volatile compound profile. The concentration of C6 volatiles, responsible for the green aroma characteristic of EVOO, decreased, while volatiles originating from the tomatoes increased. Decreases in the odour sensory attributes related to the olive fruitiness, green grass, and apple attributes were more intense in the EVOO stored with dried tomatoes at RT compared to the lower temperature, while tomato-like aromas became more pronounced at RT.

This study demonstrates that the introduction of dried tomatoes significantly alters the chemical and sensory composition of EVOO when used as a storage medium. The findings highlight the variability introduced by real storage conditions, including the addition of food products, and the resulting impact on the health, nutritional, and sensory properties of EVOO. These results provide valuable insights into the fate and preservation of EVOO’s beneficial composition and unique sensory characteristics when used in food preservation applications.

Further studies could investigate the effects of using EVOO as a storage medium for other foods to explore whether similar alterations in EVOO composition occur and whether other food items introduce new challenges or benefits in terms of oil stability and quality. Detailed mechanistic studies are needed to understand how the presence of dried tomatoes accelerates the degradation of specific phenolic compounds in EVOO, especially at higher temperatures. Identifying which specific interactions, chemical or enzymatic, are responsible could lead to solutions that help mitigate this degradation.

## Figures and Tables

**Table 1:** Free fatty acids (FFAs), peroxide value (PV), spectrophotometric indices (K_232_, K_268_, and ΔK) in fresh (0) extra virgin olive oils (EVOO) and after one (1), three (3), and six (6) months of storage with and without the addition of dry tomatoes (T) stored at room temperature (RT) and 4 °C.

Storage Time (Months)	Temp	Samples	FFA (%)	PV (meqO_2_/kg)	K_232_	K_268_	ΔK
0		EVOO	0.13 ± 0.00	9.07 ± 0.09	1.90 ± 0.09	0.13 ± 0.00	0.00 ± 0.00
1	RT	EVOO	0.11 ± 0.00 ab	7.33 ± 0.07 a	2.10 ± 0.03 a	0.13 ± 0.01	0.00 ± 0.00
		EVOO + T	0.12 ± 0.01 a	6.68 ± 0.06 b	2.04 ± 0.02 b	0.12 ± 0.00	0.00 ± 0.00
	4 °C	EVOO	0.11 ± 0.01 b	6.41 ± 0.04 c	2.03 ± 0.02 b	0.14 ± 0.01	0.00 ± 0.00
		EVOO + T	0.11 ± 0.01 b	6.14 ± 0.06 d	2.01 ± 0.01 b	0.12 ± 0.00	0.00 ± 0.00
3	RT	EVOO	0.13 ± 0.00 b	9.61 ± 0.09 a	2.46 ± 0.01 a	0.13 ± 0.01 b	0.00 ± 0.00
		EVOO + T	0.15 ± 0.01 a	8.66 ± 0.03 b	2.14 ± 0.03 b	0.13 ± 0.00 b	0.00 ± 0.00
	4 °C	EVOO	0.12 ± 0.00 b	8.29 ± 0.12 c	2.09 ± 0.07 b	0.18 ± 0.02 a	0.00 ± 0.00
		EVOO + T	0.13 ± 0.00 b	7.03 ± 0.03 d	2.02 ± 0.08 b	0.14 ± 0.00 b	0.00 ± 0.00
6	RT	EVOO	0.18 ± 0.01 b	11.39 ± 0.05 a	3.00 ± 0.09 a	0.26 ± 0.03 a	0.00 ± 0.00
		EVOO + T	0.22 ± 0.02 a	8.52 ± 0.18 b	2.19 ± 0.04 b	0.18 ± 0.01 b	0.00 ± 0.00
	4 °C	EVOO	0.16 ± 0.01 b	8.35 ± 0.08 b	2.17 ± 0.01 b	0.14 ± 0.01 b	0.00 ± 0.00
		EVOO + T	0.18 ± 0.00 b	7.50 ± 0.03 c	2.09 ± 0.05 b	0.14 ± 0.01 b	0.00 ± 0.00
EVOO *			≤0.80	≤20.0	≤2.50	≤0.22	≤0.01

Results of each parameter are expressed as mean values ± standard deviations from three independent replicates. For each storage time point (0, 1, 3, or 6), mean values marked with different lowercase letters indicate statistically significant differences (Tukey’s test, *p* < 0.05). Temp refers to storage temperature. * Current limits for the EVOO category as defined by the EEC [20].

**Table 2:** Fatty acid composition (%) in fresh (0) extra virgin olive oil (EVOO) and after one (1), three (3), and six (T6) months of storage with and without the addition of dry tomatoes (T) stored at room temperature (RT) and 4 °C.

Storage Time (Months)	Temp	Samples	Myristic (C 14:0)	Palmitic (C 16:0)	Palmitoleic (C 16:1)	Heptadecanoic (C 17:0)	Heptadecenoic (C 17:1)	Stearic (C 18:0)	Oleic (C 18:1)
0		EVOO	0.01 ± 0.00	14.84 ± 0.28	1.64 ± 0.04	0.06 ± 0.00	0.15 ± 0.00	1.77 ± 0.02	70.25 ± 0.17
1	RT	EVOO	0.01 ± 0.00	14.98 ± 0.18 a	1.67 ± 0.04	0.05 ± 0.01	0.16 ± 0.03	1.77 ± 0.02	70.03 ± 0.29
		EVOO + T	0.02 ± 0.01	15.02 ± 0.04 a	1.70 ± 0.00	0.06 ± 0.02	0.13 ± 0.02	1.91 ± 0.24	69.94 ± 0.25
	4 °C	EVOO	0.01 ± 0.00	14.95 ± 0.01 a	1.67 ± 0.02	0.07 ± 0.01	0.13 ± 0.02	1.77 ± 0.00	69.87 ± 0.02
		EVOO + T	0.01 ± 0.00	14.63 ± 0.01 b	1.68 ± 0.05	0.06 ± 0.00	0.13 ± 0.00	1.84 ± 0.05	70.23 ± 0.16
3	RT	EVOO	0.01 ± 0.00	14.55 ± 0.24 a	1.61 ± 0.03	0.05 ± 0.00	0.14 ± 0.00	1.78 ± 0.02	70.60 ± 0.14 b
		EVOO + T	0.01 ± 0.00	14.32 ± 0.07 ab	1.58 ± 0.02	0.05 ± 0.00	0.13 ± 0.00	1.80 ± 0.01	70.74 ± 0.04 ab
	4 °C	EVOO	0.01 ± 0.00	13.97 ± 0.22 b	1.59 ± 0.00	0.06 ± 0.00	0.13 ± 0.00	1.80 ± 0.00	70.98 ± 0.18 a
		EVOO + T	0.01 ± 0.00	14.72 ± 0.16 a	1.63 ± 0.03	0.06 ± 0.00	0.14 ± 0.00	1.78 ± 0.01	70.38 ± 0.17 b
6	RT	EVOO	0.01 ± 0.00	14.45 ± 0.08 a	1.58 ± 0.02	0.05 ± 0.00	0.14 ± 0.00	1.79 ± 0.01 ab	70.78 ± 0.03 bc
		EVOO + T	0.01 ± 0.00	14.61 ± 0.31 a	1.61 ± 0.04	0.06 ± 0.00	0.14 ± 0.00	1.78 ± 0.01 b	70.58 ± 0.28 c
	4 °C	EVOO	0.01 ± 0.00	13.85 ± 0.02 b	1.63 ± 0.00	0.05 ± 0.00	0.14 ± 0.00	1.79 ± 0.00 b	71.12 ± 0.00 ab
		EVOO + T	0.01 ± 0.00	13.65 ± 0.16 b	1.58 ± 0.04	0.05 ± 0.00	0.14 ± 0.00	1.80 ± 0.01 a	71.28 ± 0.16 a
		EVOO *	≤0.03	7.50–20.00	0.30–3.50	≤0.40	≤0.60	0.50–5.00	55.00–83.00
**Storage Time** **(months)**	**Temp**	**Samples**	**Linoleic** **(C 18:2)**	**Linolenic (C18:3)**	**Arachidic** **(C 20:0)**	**Eicosenoic** **(C 20:1)**	**Behenic** **(C 22:0)**	**Eicosenoic Acid** **(C 22:1)**	**Lignoceric** **(C 24:0)**
0		EVOO	9.68 ± 0.10	0.83 ± 0.01	0.32 ± 0.01	0.30 ± 0.01	0.10 ± 0.00	0.00 ± 0.00	0.05 ± 0.00
1	RT	EVOO	9.70 ± 0.10	0.83 ± 0.00 ab	0.32 ± 0.02 b	0.32 ± 0.01	0.11 ± 0.02	0.00 ± 0.00	0.05 ± 0.01
		EVOO + T	9.68 ± 0.10	0.80 ± 0.03 b	0.32 ± 0.01 ab	0.29 ± 0.03	0.09 ± 0.01	0.00 ± 0.00	0.05 ± 0.01
	4 °C	EVOO	9.84 ± 0.07	0.87 ± 0.03 a	0.31 ± 0.00 b	0.35 ± 0.03	0.10 ± 0.01	0.00 ± 0.00	0.05 ± 0.00
		EVOO + T	9.76 ± 0.05	0.84 ± 0.01 ab	0.35 ± 0.02 a	0.29 ± 0.03	0.10 ± 0.00	0.00 ± 0.00	0.05 ± 0.00
3	RT	EVOO	9.63 ± 0.07	0.82 ± 0.01	0.34 ± 0.00	0.31 ± 0.02	0.10 ± 0.01	0.00 ± 0.00	0.05 ± 0.01
		EVOO + T	9.70 ± 0.02	0.83 ± 0.01	0.34 ± 0.00	0.33 ± 0.01	0.11 ± 0.00	0.00 ± 0.00	0.05 ± 0.00
	4 °C	EVOO	9.77 ± 0.05	0.83 ± 0.01	0.34 ± 0.00	0.33 ± 0.00	0.11 ± 0.00	0.00 ± 0.00	0.06 ± 0.00
		EVOO + T	9.67 ± 0.00	0.82 ± 0.00	0.33 ± 0.01	0.30 ± 0.01	0.11 ± 0.00	0.00 ± 0.00	0.05 ± 0.01
6	RT	EVOO	9.63 ± 0.05 b	0.79 ± 0.00	0.33 ± 0.01	0.29 ± 0.00 b	0.10 ± 0.00 ab	0.00 ± 0.00	0.05 ± 0.00
		EVOO + T	9.63 ± 0.04 b	0.80 ± 0.01	0.33 ± 0.01	0.30 ± 0.01 b	0.10 ± 0.00 ab	0.00 ± 0.00	0.05 ± 0.01
	4 °C	EVOO	9.82 ± 0.01 a	0.81 ± 0.00	0.33 ± 0.00	0.30 ± 0.01 b	0.10 ± 0.00 b	0.00 ± 0.00	0.05 ± 0.00
		EVOO + T	9.84 ± 0.01 a	0.82 ± 0.01	0.34 ± 0.01	0.32 ± 0.00 a	0.11 ± 0.00 a	0.00 ± 0.00	0.05 ± 0.01
		EVOO *	2.50–21.00	≤1.00	≤0.60	≤0.50	≤0.20	/	≤0.20
**Storage Time** **(Months)**	**Temp**	**Samples**	**∑SFA**		**∑MUFA**		**∑PUFA**	**Oleic/Linoleic Ratio (C18:1/C18:2)**	
0		EVOO	17.1 ± 0.3		72.4 ± 0.1		10.5 ± 0.1	7.26 ± 0.06	
1	RT	EVOO	17.3 ± 0.1 ab		72.2 ± 0.2		10.5 ± 0.1 ab	7.22 ± 0.10	
		EVOO + T	17.5 ± 0.2 a		72.1 ± 0.3		10.5 ± 0.1 b	7.22 ± 0.10	
	4 °C	EVOO	17.3 ± 0.0 ab		72.0 ± 0.0		10.7 ± 0.0 a	7.10 ± 0.05	
		EVOO + T	17.1 ± 0.1 b		72.3 ± 0.1		10.6 ± 0.1 ab	7.20 ± 0.05	
3	RT	EVOO	16.8 ± 0.2 ab		72.8 ± 0.1 ab		10.5 ± 0.1	7.32 ± 0.04	
		EVOO + T	16.7 ± 0.1 ab		72.8 ± 0.0 ab		10.5 ± 0.0	7.29 ± 0.01	
	4 °C	EVOO	16.4 ± 0.2 b		73.0 ± 0.2 a		10.6 ± 0.1	7.27 ± 0.01	
		EVOO + T	17.1 ± 0.1 a		72.5 ± 0.1 b		10.5 ± 0.0	7.28 ± 0.01	
6	RT	EVOO	16.8 ± 0.1 a		72.8 ± 0.1 b		10.4 ± 0.1 b	7.35 ± 0.03 a	
		EVOO + T	16.9 ± 0.3 a		72.6 ± 0.2 b		10.4 ± 0.1 b	7.33 ± 0.01 a	
	4 °C	EVOO	16.2 ± 0.0 b		73.2 ± 0.0 a		10.6 ± 0.0 a	7.25 ± 0.01 b	
		EVOO + T	16.0 ± 0.1 b		73.3 ± 0.1 a		10.7 ± 0.0 a	7.24 ± 0.01 b	

Results of each fatty acid are expressed as mean values ± standard deviations from three independent replicates. For each storage time point (0, 1, 3, or 6), mean values marked with different lowercase letters indicate statistically significant differences (Tukey’s test, *p* < 0.05). Temp refers to storage temperature; SFA—saturated fatty acid; MUFA—monounsaturated fatty acid; PUFA—polyunsaturated fatty acid. * Current limits for the EVOO category as defined by the EEC [20].

**Table 3:** The concentration (mg/kg) of phenolic compounds in fresh (0) extra virgin olive oil (EVOO) and after one (1), three (3), and six (6) months of storage with and without the addition of dry tomatoes (T) stored at room temperature (RT) and 4 °C.

Storage Time (Months)	Temp	Samples	Simple Phenols						Phenolic Acids	
			Tyrosol	Hydroxytyrosol	Hydroxytyrosol Acetate		Vanillin		*p*-Coumaric Acid	Vanillic Acid
0		EVOO	4.01 ± 0.04	2.55 ± 0.09	0.12 ± 0.00		0.17 ± 0.00		1.63 ± 0.03	0.49 ± 0.00
1	RT	EVOO	3.39 ± 0.10 b	2.23 ± 0.09 b	0.10 ± 0.00 c		0.15 ± 0.01		1.41 ± 0.08 b	0.42 ± 0.03 b
		EVOO + T	3.34 ± 0.25 b	1.46 ± 0.20 c	1.28 ± 0.09 a		0.17 ± 0.01		1.56 ± 0.07 ab	0.56 ± 0.02 a
	4 °C	EVOO	4.24 ± 0.03 a	2.85 ± 0.11 a	0.11 ± 0.00 c		0.16 ± 0.01		1.60 ± 0.04 a	0.48 ± 0.01 b
		EVOO + T	3.87 ± 0.18 a	2.19 ± 0.12 b	0.40 ± 0.03 b		0.16 ± 0.01		1.48 ± 0.08 ab	0.43 ± 0.03 b
3	RT	EVOO	3.63 ± 0.25 a	2.24 ± 0.07 a	0.16 ± 0.01 c		0.17 ± 0.02		1.64 ± 0.22	0.48 ± 0.03 a
		EVOO + T	4.24 ± 0.17 a	2.12 ± 0.16 a	1.83 ± 0.12 a		0.18 ± 0.02		1.51 ± 0.10	0.32 ± 0.01 b
	4 °C	EVOO	3.94 ± 0.09 a	1.89 ± 0.12 a	0.13 ± 0.02 c		0.16 ± 0.03		1.68 ± 0.27	0.46 ± 0.06 a
		EVOO + T	2.90 ± 0.35 b	1.31 ± 0.21 b	0.59 ± 0.10 b		0.17 ± 0.01		1.49 ± 0.09	0.43 ± 0.04 a
6	RT	EVOO	4.15 ± 0.02 a	2.99 ± 0.03 a	0.13 ± 0.01 b		0.15 ± 0.00		1.48 ± 0.03 b	0.43 ± 0.01 a
		EVOO + T	3.28 ± 0.17 b	0.77 ± 0.03 c	2.01 ± 0.08 a		0.15 ± 0.01		1.79 ± 0.17 a	0.19 ± 0.02 b
	4 °C	EVOO	4.02 ± 0.25 a	2.88 ± 0.15 a	0.11 ± 0.01 b		0.15 ± 0.02		1.45 ± 0.05 b	0.43 ± 0.03 a
		EVOO + T	3.45 ± 0.21 b	1.98 ± 0.22 b	0.63 ± 0.03 b		0.16 ± 0.01		1.38 ± 0.13 b	0.43 ± 0.03 a
**Storage Time** **(Months)**	**Temp**	**Samples**	**Flavonoids**				**Lignans**			
			**Luteolin**		**Apigenin**		**Pinoresinol**		Acetoxypinoresinol *	
0		EVOO	2.56 ± 0.03		0.53 ± 0.02		6.83 ± 0.05		11.8 ± 0.4	
1	RT	EVOO	1.98 ± 0.03 ab		0.41 ± 0.01 b		5.68 ± 0.29 b		10.1 ± 0.5 b	
		EVOO + T	1.59 ± 0.14 b		0.65 ± 0.01 a		6.77 ± 0.07 a		12.0 ± 0.8 a	
	4 °C	EVOO	2.49 ± 0.34 a		0.51 ± 0.09 ab		6.89 ± 0.39 a		11.5 ± 0.9 ab	
		EVOO + T	2.06 ± 0.18 ab		0.53 ± 0.05 ab		6.42 ± 0.30 ab		10.9 ± 0.5 ab	
3	RT	EVOO	2.40 ± 0.16 a		0.49 ± 0.03 ab		7.26 ± 0.73 a		12.6 ± 0.9	
		EVOO + T	0.85 ± 0.15 c		0.40 ± 0.03 b		5.60 ± 0.07 b		10.5 ± 0.2	
	4 °C	EVOO	2.69 ± 0.12 a		0.56 ± 0.02 a		7.37 ± 0.64 a		12.6 ± 1.7	
		EVOO + T	1.96 ± 0.15 b		0.52 ± 0.06 a		6.64 ± 0.60 ab		11.3 ± 0.5	
6	RT	EVOO	2.47 ± 0.10 a		0.56 ± 0.03 a		6.16 ± 0.12 a		11.8 ± 0.3 a	
		EVOO + T	0.44 ± 0.06 b		0.36 ± 0.03 b		5.12 ± 0.09 b		8.39 ± 0.50 c	
	4 °C	EVOO	1.56 ± 0.95 ab		0.49 ± 0.14 ab		6.03 ± 0.24 a		10.4 ± 0.5 b	
		EVOO + T	1.80 ± 0.27 a		0.52 ± 0.05 ab		6.03 ± 0.42 a		11.3 ± 0.5 ab	
**Storage Time** **(Months)**	**Temp**	**Samples**	**Secoiridoids**							
			Oleuropein + Ligstroside Aglycones I & II *	Ligstroside Aglycon (Isomer II) *	Oleochantal (*p*-HPEA-EDA) *	Oleuropein Aglycone (Isomer I) *		Oleuropein Aglycone (Isomer II) *	Oleuropein Aglycone (Isomer III) *	Oleacein (3,4-DHPEA-EDA) *
0		EVOO	19.6 ± 1.0	11.1 ± 0.6	50.1 ± 1.6	44.9 ± 1.2		48.8 ± 0.9	13.8 ± 0.25	93.6 ± 3.0
1	RT	EVOO	14.7 ± 0.5 ab	8.87 ± 0.79	39.4 ± 1.4 b	35.7 ± 1.3 b		40.2 ± 0.5 c	11.8 ± 0.1 b	75.0 ± 3.0 b
		EVOO + T	14.4 ± 1.8 b	9.12 ± 0.43	34.3 ± 0.7 c	26.4 ± 1.8 c		50.1 ± 0.9 a	15.4 ± 0.9 a	28.3 ± 2.1 d
	4 °C	EVOO	17.8 ± 1.1 a	11.2 ± 1.4	46.3 ± 1.1 a	42.3 ± 1.5 a		47.1 ± 2.2 ab	13.3 ± 1.5 ab	85.6 ± 2.5 a
		EVOO + T	15.6 ± 1.0 ab	9.66 ± 0.59	39.0 ± 1.61 b	33.8 ± 1.8 b		44.1 ± 0.8 b	13.1 ± 0.6 ab	54.1 ± 3.4 c
3	RT	EVOO	14.4 ± 3.2	11.1 ± 0.7 a	39.5 ± 2.0 a	35.3 ± 1.5 b		39.8 ± 1.8 b	12.8 ± 1.0	71.0 ± 3.5 a
		EVOO + T	13.0 ± 1.5	5.18 ± 1.19 b	19.5 ± 0.5 c	21.6 ± 1.8 c		36.0 ± 1.2 b	12.6 ± 1.1	9.51 ± 1.1 c
	4 °C	EVOO	13.9 ± 1.2	12.8 ± 0.9 a	40.3 ± 2.9 a	42.3 ± 3.5 a		38.0 ± 1.8 b	11.0 ± 1.3	75.7 ± 5.1 a
		EVOO + T	16.8 ± 1.8	9.82 ± 1.27 a	32.7 ± 0.5 b	38.0 ± 0.6 ab		46.0 ± 2.6 a	10.4 ± 0.6	45.9 ± 2.1 b
6	RT	EVOO	12.6 ± 0.6 b	10.8 ± 1.0 a	40.0 ± 1.9 a	33.7 ± 0.9 b		39.5 ± 2.1 a	12.8 ± 0.7 a	71.5 ± 3.1 a
		EVOO + T	9.92 ± 0.53 c	5.55 ± 0.66 b	11.3 ± 2.4 c	18.8 ± 1.0 c		29.6 ± 1.1 b	8.68 ± 0.54 b	8.83 ± 1.21 c
	4 °C	EVOO	13.3 ± 1.4 ab	9.79 ± 1.00 a	37.8 ± 0.5 ab	40.7 ± 0.7 a		34.7 ± 0.5 ab	9.58 ± 0.71 b	71.6 ± 0.2 a
		EVOO + T	15.4 ± 0.5 a	9.05 ± 0.55 a	31.7 ± 4.1 b	32.7 ± 1.1 b		36.3 ± 4.3 a	11.6 ± 1.0 a	38.0 ± 2.2 b

Results of each phenolic compound are expressed as mean values ± standard deviations from three independent replicates. For each storage time point (0, 1, 3, or 6), mean values marked with different lowercase letters indicate statistically significant differences (Tukey’s test, *p* < 0.05). * The phenolic compounds were quantified semi-quantitatively by expressing concentrations as equivalents of hydroxytyrosol for hydroxytyrosol acetate, oleuropein for secoiridoids, and pinoresinol for acetoxypinoresinol, assuming a response factor of 1. Temp refers to storage temperature.

**Table 4:** The concentrations (mg/kg) of volatile compounds in fresh (0) extra virgin olive oil (EVOO) and after one (1) and six (6) months of storage with and without the addition of dry tomatoes (T) stored at room temperature (RT) and 4 °C.

Storage Time (Months)	Temp	Samples	C5 Volatiles							
			3—Pentanone		1-Pentene-3-one	(*E*)-2-Penten-1-ol	(*Z*)-2-Pentenal *	(*E*)-2-Pentenal	Total C5 Volatiles	
0		EVOO	0.095 ± 0.002		1.24 ± 0.01	0.020 ± 0.002	0.031 ± 0.001	0.073 ± 0.000	1.45 ± 0.01	
1	RT	EVOO	0.098 ± 0.002 a		1.28 ± 0.01 a	0.023 ± 0.000 a	0.027 ± 0.001 a	0.074 ± 0.002 a	1.51 ± 0.01 a	
		EVOO + T	0.071 ± 0.001 b		0.742 ± 0.000 d	0.016 ± 0.000 b	0.013 ± 0.000 c	0.062 ± 0.003 b	0.905 ± 0.003 d	
	4 °C	EVOO	0.094 ± 0.003 a		1.22 ± 0.01 b	0.021 ± 0.000 a	0.030 ± 0.001 a	0.072 ± 0.000 a	1.44 ± 0.01 b	
		EVOO + T	0.075 ± 0.001 b		0.938 ± 0.013 c	0.019 ± 0.001 b	0.021 ± 0.001 b	0.064 ± 0.001 b	1.12 ± 0.02 c	
6	RT	EVOO	0.083 ± 0.001 a		0.851 ± 0.030 a	0.045 ± 0.021	0.015 ± 0.001 a	0.062 ± 0.000 a	1.06 ± 0.05 a	
		EVOO + T	0.064 ± 0.001 b		0.149 ± 0.003 c	0.012 ± 0.000	0.003 ± 0.000 c	0.039 ± 0.000 d	0.266 ± 0.004 c	
	4 °C	EVOO	0.074 ± 0.004 a		0.907 ± 0.018 a	0.053 ± 0.001	0.016 ± 0.000 a	0.059 ± 0.000 b	1.11 ± 0.021 a	
		EVOO + T	0.061 ± 0.002 b		0.558 ± 0.009 b	0.016 ± 0.002	0.008 ± 0.001 b	0.046 ± 0.000 c	0.690 ± 0.008 b	
**Storage Time** **(Months)**	**Temp**	**Samples**	**C6 Volatiles**							
			**Hexanal**		**(*E*)-2-Hexenal**	(*Z*)-2-Hexenal *	(*E*)-3-Hexenal *	(*Z*)-3-Hexenal *	**Total C6 Aldehydes**	
0		EVOO	0.873 ± 0.001		44.6 ± 0.15	0.402 ± 0.004	0.140 ± 0.001	0.226 ± 0.002	46.2 ± 0.2	
1	RT	EVOO	0.969 ± 0.016 a		46.0 ± 0.7 a	0.381 ± 0.028 ab	0.135 ± 0.003 a	0.171 ± 0.000 b	47.7 ± 0.8 a	
		EVOO + T	0.759 ± 0.015 b		35.8 ± 0.5 c	0.325 ± 0.010 b	0.106 ± 0.001 c	0.104 ± 0.002 c	37.1 ± 0.5 c	
	4 °C	EVOO	0.929 ± 0.012 a		43.8 ± 0.3 a	0.417 ± 0.007 a	0.142 ± 0.000 a	0.237 ± 0.003 a	45.5 ± 0.3 a	
		EVOO + T	0.814 ± 0.013 b		39.0 ± 0.9 b	0.352 ± 0.025 ab	0.118 ± 0.001 b	0.162 ± 0.004 b	40.5 ± 0.9 b	
6	RT	EVOO	1.043 ± 0.100 a		35.6 ± 0.6 a	0.197 ± 0.022 b	0.063 ± 0.001 b	0.044 ± 0.002 b	36.9 ± 0.5 a	
		EVOO + T	0.742 ± 0.001 b		19.3 ± 0.1 c	0.164 ± 0.004 b	0.050 ± 0.001 c	0.018 ± 0.000 c	20.2 ± 0.1 c	
	4 °C	EVOO	0.794 ± 0.011 ab		35.7 ± 0.9 a	0.305 ± 0.001 a	0.082 ± 0.002 a	0.125 ± 0.010 a	37.0 ± 0.9 a	
		EVOO + T	0.838 ± 0.082 ab		29.8 ± 0.4 b	0.208 ± 0.002 b	0.064 ± 0.002 b	0.061 ± 0.003 b	31.0 ± 0.3 b	
**Storage Time** **(Months)**	**Temp**	**Samples**	**C6 Volatiles**							
			**1-Hexanol**	**(*E*)-3-Hexen-1-ol**	**(*Z*)-3-Hexen-1-ol**	**(*E*)-2-Hexen-1-ol**	**(*Z*)-2-Hexen-1-ol**	**Total C6 Alcohols**	**Hexyl Acetate**	**Total C6 Volatiles**
0		EVOO	0.942 ± 0.006	0.021 ± 0.000	1.19 ± 0.01	1.18 ± 0.01	0.011 ± 0.000	3.34 ± 0.03	0.018 ± 0.011	49.6 ± 0.2
1	RT	EVOO	0.969 ± 0.008 a	0.021 ± 0.000 a	1.22 ± 0.01 a	1.22 ± 0.02 a	0.011 ± 0.000 a	3.44 ± 0.04 a	0.011 ± 0.002 b	51.1 ± 0.8 a
		EVOO + T	0.759 ± 0.001 b	0.017 ± 0.003 ab	0.904 ± 0.004 d	0.926 ± 0.027 c	0.008 ± 0.001 b	2.61 ± 0.03 d	0.021 ± 0.000 a	39.8 ± 0.5 c
	4 °C	EVOO	0.921 ± 0.039 a	0.020 ± 0.002 a	1.18 ± 0.01 b	1.16 ± 0.01 a	0.010 ± 0.000 a	3.29 ± 0.03 b	0.012 ± 0.003 b	48.8 ± 0.2 a
		EVOO + T	0.824 ± 0.016 b	0.012 ± 0.000 b	1.00 ± 0.00 c	1.01 ± 0.02 b	0.009 ± 0.000 a	2.86 ± 0.04 c	0.019 ± 0.000 a	43.3 ± 0.9 b
6	RT	EVOO	0.782 ± 0.026 a	0.015 ± 0.000 a	1.12 ± 0.03 ab	0.999 ± 0.043 a	0.006 ± 0.001 ab	2.92 ± 0.10 a	0.008 ± 0.002	39.8 ± 0.6 a
		EVOO + T	0.630 ± 0.005 b	0.012 ± 0.000 b	0.855 ± 0.048 c	0.778 ± 0.004 b	0.014 ± 0.002 a	2.29 ± 0.06 b	0.018 ± 0.002	22.5 ± 0.2 c
	4 °C	EVOO	0.801 ± 0.026 a	0.015 ± 0.000 a	1.15 ± 0.03 a	1.06 ± 0.04 a	0.005 ± 0.001 b	3.03 ± 0.10 a	0.009 ± 0.000	40.0 ± 1.0 a
		EVOO + T	0.702 ± 0.002 b	0.013 ± 0.000 b	1.003 ± 0.005 b	0.862 ± 0.028 b	0.003 ± 0.004 b	2.58 ± 0.04 b	0.016 ± 0.004	33.6 ± 0.3 b

Results for each volatile compound are expressed as mean values ± standard deviations from three independent replicates. For each storage time point (1 or 6 months), mean values marked with different lowercase letters indicate statistically significant differences (Tukey’s test, *p* < 0.05). * The volatile compounds quantified semi-quantitatively by expressing concentrations (mg/kg) as equivalents of structurally similar compounds, assuming a response factor of 1.

## Data Availability

Data are contained within the article.

## Supplementary Materials

The following supporting information can be downloaded at: https://www.mdpi.com/article/10.3390/molecules29235497/s1, Figure S1: High performance liquid chromatography with diode-array detection chromatograms of phenolic compounds used for calibration curves, monitored at λ 280 nm (a), λ 320 nm (b), and λ 365 nm (c).

## Abbreviations

ANOVA—analysis of variance; DPPH—2,2-diphenyl-1-picrylhydrazyl; EVOO—extra virgin olive oil; FAMEs—fatty acid methyl esters; FFAs—free fatty acids; GC-FID—gas chromatography with flame ionization detector; HPLC-DAD—high-performance liquid chromatography with diode-array detection; HS-SPME—headspace solid-phase microextraction; IB—Istarska bjelica; IOC—the International Olive Council; K_232_, K_268_, and ΔK—spectrophotometric indices; MUFA—monounsaturated fatty acid; PUFA—polyunsaturated fatty acid; PV—peroxide value; RSA—radical-scavenging activity; RT—room temperature; T—temperature; TE—Trolox equivalent; TIPCs—total identified phenolic compounds.
