# Supplementary material for: Changes in the Composition, Antioxidant Activity, and Sensory Attributes of Olive Oil Used as a Storage Medium for Dried Tomato Preservation
Source: Molecules. 2024 Nov 21;29(23):5497. doi: 10.3390/molecules29235497 (PMC11643605; doi:10.3390/molecules29235497)
Supplement: Supplementary file 1 [file molecules-29-05497-s001.zip › molecules-3265604-supplementary.pdf]

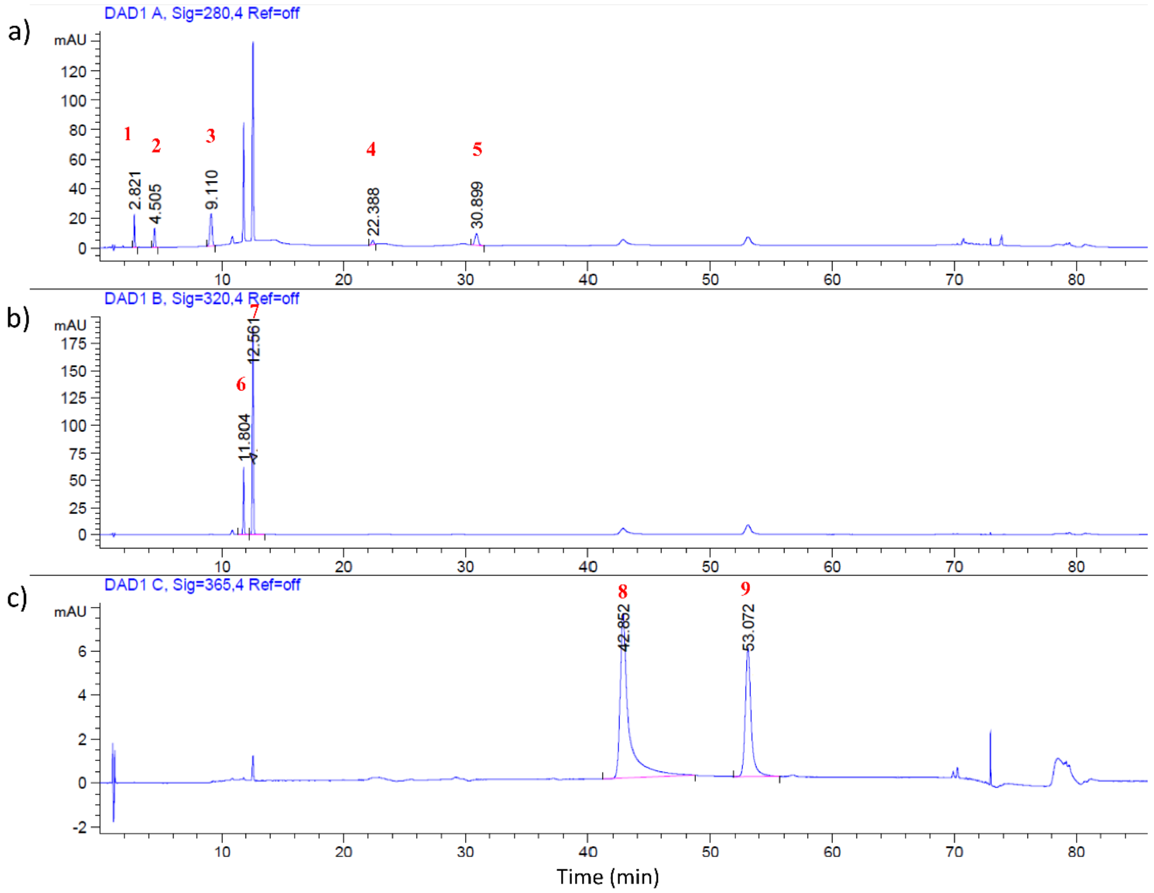

Figure S1. High performance liquid chromatography with diode-array detection chromatograms of phenolic compounds used for calibration curves, monitored at: λ 280 nm (**a**), λ 320 nm (**b**), and λ 365 nm (**c**). Peak identification: 1, hydroxytyrosol; 2, tyrosol; 3, vanillic acid; 4. oleuropein; 5, pinoresinol; 6, vanillin; 7, *p*-coumaric acid; 8, luteolin; and 9, apigenin.
